# Conservation Genomics of Wild Red Sage (*Salvia miltiorrhiza*) and Its Endangered Relatives in China: Population Structure and Interspecific Relationships Revealed From 2b-RAD Data

**DOI:** 10.3389/fgene.2021.688323

**Published:** 2021-05-11

**Authors:** Xuan Zhou, Zhi-Cheng Zhang, Yan-Bo Huang, Han-Wen Xiao, Jun-Jie Wu, Zhe-Chen Qi, Yu-Kun Wei

**Affiliations:** ^1^Zhejiang Province Key Laboratory of Plant Secondary Metabolism and Regulation, College of Life Sciences and Medicine, Zhejiang Sci-Tech University, Hangzhou, China; ^2^Shanghai Key Laboratory of Plant Functional Genomics and Resources and Eastern China Conservation Center for Wild Endangered Plant Resources, Shanghai Chenshan Botanical Garden, Shanghai, China; ^3^Shaoxing Academy of Biomedicine of Zhejiang Sci-Tech University, Shaoxing, China

**Keywords:** *Salvia miltiorrhiza*, 2b-RAD, population genomics, conservation, genetic diversity, *Salvia bowleyana*, *Salvia paramiltiorrhiza*, wild germplasm

## Abstract

Red sage (*Salvia miltiorrhiza*) is a widely used medicinal plant for treatment of cardiovascular and cerebrovascular diseases. Because of excessive excavation by huge market demand and habitat loss by human activities, the wild population resources of *S. miltiorrhiza* have reduced drastically in recent years. Meanwhile, population status of two closely related species *S. bowleyana* and *S. paramiltiorrhiza* were in a trend of decreasing due to their potential replacement of *S. miltiorrhiza*. Particularly, *S. paramiltiorrhiza* was threatened and endemic to a small region in eastern China. However, to date there has been no conservation genetic research reported for wild *S. miltiorrhiza* population and its endangered relatives. Assess the wild germplasm diversity for *S. miltiorrhiza* and its related species would provide fundamental genetic background for cultivation and molecular breeding of this medicinally important species. In the present study, we investigated the genetic diversity, population structure, and intra/inter-specific differentiation of *S. miltiorrhiza* and above two relatives using 2b-RAD genome-wide genotyping method. By investigating 81 individuals of *S. miltiorrhiza*, 55 individuals of *S. bowleyana* and 15 individuals of *S. paramiltiorrhiza* from 23 locations in China, we obtained 23,928 SNPs in total. A comparatively high genetic diversity was observed in *S. miltiorrhiza* (π = 0.0788, *H*_*e*_ = 0.0783 ± 0.0007). The observed and expected heterozygosity in populations of these three species ranged from 0.0297 to 0.1481 and 0.0251 to 0.831, respectively. Two major lineage groups were detected in the examined *S. miltiorrhiza* populations. The results indicated that Dabie Mountain as a genetic diversity center of *S. miltiorrhiza* and possible complex inter-specific genetic exchange/hybridization occurred between *S. miltiorrhiza* and the two relatives. We suggest that strategic conservation and germplasm preservation should be considered not only for wild populations of *S. miltiorrhiza*, but also for its related *S. bowleyana* and *S. paramiltiorrhiza.*

## Introduction

Medicinal plants are invaluable resource of maintaining health for the majority of world’s population. At present, more than 80% of the world’s population depends on herbal medicine for primary healthcare needs, while millions gain income from their wild harvest or cultivation, or are involved in their trading or processing (Hamilton, 2004). With the growing human populations, the demand for medicinal plants kept growing rapidly throughout the world for the past decades for their usage in herbal drugs and natural health products (Bentley, 2010; Jamshidi-Kia et al., 2018). According to IUCN, 50,000 and 80,000 flowering plant species are estimated to be used for pharmaceutical purposes around the world. For instance, China and India have the highest numbers of medicinal plants used, with 11,146 and 7,500 species, respectively. Among these numbers, 90% species were directly harvested from wild resources (Sarwat et al., 2011; Chen et al., 2016). Overexploitation and habitat destruction by human activity has led to an alarming number of medicinal plant species under threat of extinction. About 15,000 species are exposed to a risk of extinction due to high harvesting and destruction of habitats. A high proportion of medicinal plant species have become endangered in some species rich areas, threatening the regional biodiversity and ecosystem stability, especially in developing countries (Heywood and Iriondo, 2003; Hamilton, 2008; Bentley, 2010; Jamshidi-Kia et al., 2018).

One way to relief this situation is domestic cultivation. A number of governments and agencies are recommending that wild medicinal species should be brought into cultivation systems (WHO et al., 1993). Increased cultivation of medicinal plant is not only a means for meeting the huge market demands for drug and herbal remedies based on medicinal plant, but also a way to decrease harvest volume on wild populations, benefiting to the recovery of their wild resources (Schippmann et al., 2002; Canter et al., 2005). However, medicinal plant production through cultivation would probably result in genetic diversity reduce of cultivars due to founder effect and artificial selection, resembling the genetic bottleneck during the domestication of cereal species (Doebley et al., 2006). Decreased genetic diversity will lead them susceptible to pests, diseases, abiotic stress. Meanwhile, cultivation could also negatively affect the incentives to conserve wild populations (Schippmann et al., 2006). Wild population and relatives with genetic resources have proven to be very valuable in agricultural breeding practice (Tyack et al., 2020). The conservation of wild populations could maintain genetic diversity and allows for the continuing evolution of the gene pool as a germplasm resource for future crop improvement (Tyack et al., 2020; Vining et al., 2020). In this regard, systematic collection and analyses of genetic diversity and relationships in wild medicinal plant populations can lay important foundations for the conservation of species as well as designing strategies for development of commercially relevant varieties (Govindaraj et al., 2015).

**FIGURE 1:**
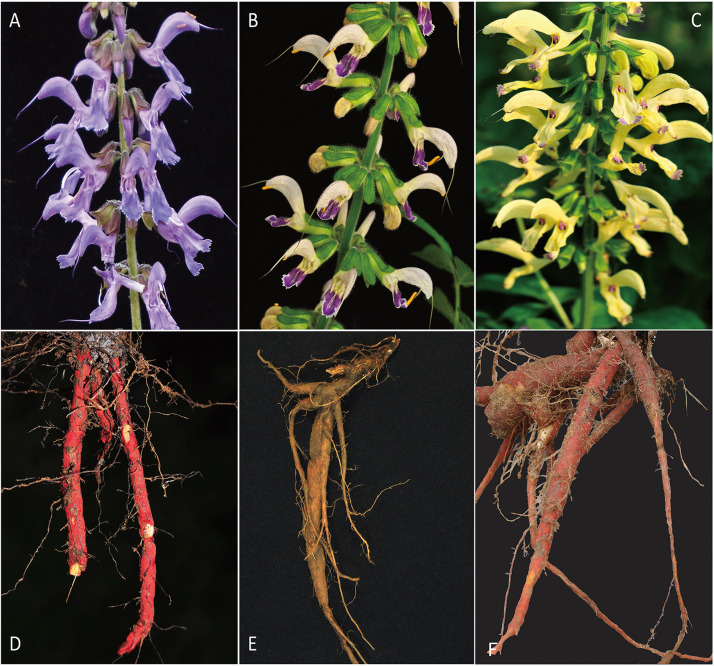
Flower and root morphology the three studied species. **(A)** flower of *S. miltiorrhiza*. **(B)** flower of *S. bowleyana*. **(C)** flower of *S. paramiltiorrhiza.* **(D)** root of *S. miltiorrhiza*. **(E)** root of *S. bowleyana*. **(F)** root of *S. paramiltiorrhiza*.

*Salvia miltiorrhiza* Bunge (Lamiaceae), the red sage, is an indigenous medicinal herb in China. Wild populations of *S. miltiorrhiza* usually grows on the hillsides, stream banks and understories in broad-leaved deciduous forests in central and eastern China. Its dried roots, commonly known as Danshen, have long been used in traditional Chinese medicine for treatment of cardiovascular and cerebrovascular diseases (Wang, 2010; Zhang et al., 2014). The production of *S. miltiorrhiza* in China achieved a total revenue of around 75 million USD in 2015 (Kum, 2019). Due to the huge market demand, although mostly harvested from cultivation, the wild populations of *S. miltiorrhiza* reduced drastically in recent years by excessive excavation and habitat destruction (Yang et al., 2017). Moreover, germplasm depression was frequently observed in cultivated *S. miltiorrhiza.* Meanwhile, two closely related species *S. bowleyana* Dunn and *S. paramiltiorrhiza* Li and Huang are also under a pressure of population decrease as frequent substitutes to *S. miltiorrhiza* (Field observation in the past decade). Particularly, populations of *S. paramiltiorrhiza* was critically threatened and can only be found in restricted areas in Hubei and Anhui, China (Huang and Li, 1981; Hao et al., 2015). Morphologically, these three species can be distinguished from each other mainly by their flower and root colors (Figure 1). However, previous phylogenetic studies based on plastid DNA and a few nuclear loci showed that their evolutionary relationships remain unresolved (Li et al., 2013; Hu et al., 2018). As wild populations and relatives as germplasm resources may provide infinite potentials for superior breeding varieties to improve the quality of cultivars (Vining et al., 2020; Wang et al., 2020), it is essential to conserve and assess the genetic diversity of *S. miltiorrhiza* and its relatives in natural populations.

Molecular markers are playing a significant role in studying population genetics of medicinal plants (Sarwat et al., 2011; Wang et al., 2020). With suitable genetic makers, we can understand the germplasm diversity, such as single nucleotide polymorphism (SNP), allele frequencies, extend and distribution of genetic diversity, and population structure, under a rigorous mathematical framework. Such information gained can be then referred to devise a proper conservation strategy and genebank management. With its ultra-high-throughput, next-generation sequencing (NGS) technology could provide an efficient toolkit in producing large amount of SNP markers for organisms through simplified genome sequencing, for example, restriction site-associated DNA sequencing (RADSeq), genotyping-by-sequencing (GBS) or other related techniques (Davey et al., 2011; Peterson et al., 2012; Andrews et al., 2016). 2b-RAD, characterized by producing uniform fragments generated by type IIB restriction endonuclease, is a flexible and cost-effective method outperform other RAD-seq methods with higher markers numbers, repeatability, and genomic coverage density (Wang et al., 2012).

In present study, we assessed the genetic diversity of *S. miltiorrhiza* and two closely related species (*S. bowleyana* and *S. paramiltiorrhiza*) using 2b-RAD sequencing. We investigated the phylogenetic relationships and several aspects of the population genetics of focusing on wild *S. miltiorrhiza* populations including genetic diversity, population structure, and differentiation, as well as estimation of demographic history. We hope the methods and results presented here could be useful in guidance of wild germplasm conservation and management for *S. miltiorrhiza* and other medicinal plants in the future.

## Materials and Methods

### Sampling and DNA Extraction

A total of 151 individuals from 23 sites in central and eastern China were sampled and analyzed in this study, including *S. miltiorrhiza* (81 individuals from 13 wild populations), *S. bowleyana* (55 individuals from 9 wild populations) and *S. paramiltiorrhiza* (15 individuals from 1 wild populations) (Supplementary Table 1). Our sampling focused on an area where populations of all three species occur, in order to assess their potential gene flows. Leaves collected from each individual were dried in silica gel. Genomic DNA was extracted from dried leaf powder using a modified CTAB method (Allen et al., 2006). First, gel-dried leaf samples were put into liquid nitrogen and quickly ground to powder; the powder was then immediately transferred into a clean 1.5 ml microcentrifuge tube and mixed immediately with 600 μl 2 × CTAB extracting solution and incubated in a water bath at 65°C for 45 min. The samples were then centrifuged twice at 12,000 rpm for 10 min, after which the supernatant was transferred to another clean 1.5 ml microcentrifuge tube. Then 5 μl DNase free RNase (10 μg/μl) was added and incubate at 37°C for 15 min, intermittently mixed up for 2–3 times. The same volume of chloroform-isoamyl alcohol (24:1) was then added and mixed up the phases by gentle inversion. The sample was centrifuged at 12,000 rpm for 5 min, after which the supernatant was transferred to a new 1.5 ml centrifuge tube and mixed with the same volume of isopropanol, then incubated at −20°C for about 1 h. The solution was centrifuged at 12,000 rpm for 10 min, then the supernatant liquor was carefully discarded. The DNA was then cleaned twice with 75% ethanol and air dried at room temperature. The cleaned DNA was then dissolved in 50 μl TE. DNA quantity was assessed with 1% agarose gel electrophoresis, and Qubit 3.0 fluorescent quantitative assay and NanoVue were used to test purity and density.

### Multi-IsoRAD Sequencing and SNP Calling

Library construction and sequencing were conducted following Wang et al. (2016), an updated 2b-RAD approach that allows the preparation of five concatenated isoRAD tags for Illumina paired-end sequencing. After DNA extraction and quality control, the high-quality genomic DNA (600 ng) was digested with restriction enzyme of IIB type (*Bsa*XI) at 37° for 1 h, followed by enzyme heat inactivation at 65° for 20 min. The ligation reaction was performed by combining 5 μl of digested DNA with 20 μl of a ligation master mix containing 0.4 μM each of library-specific adaptors with fully degenerate cohesive ends (5′ -NNN- 3′), 0.2 mM adenosine 5′-triphosphate (New England Biolabs), and 1000 U T4 DNA ligase. Then PCR amplification was performed. Ligation was carried out at 16° for 3 h, with subsequent heat inactivation for 10 min at 65°. In Multi-isoRAD protocol, the single-tag constructs can be further digested by *Sap*I to generate distinct cohesive ends and then ligated in a predefined order to produce five concatenated tags for Illumina paired-end sequencing using modified adaptors and biotin-labeled primers. After individual libraries were pooled into equimolar amounts, the pool quality was verified on Agilent 2100 Bioanalyzer. The paired-end RAD-seq were generated using Illumina Hiseq X ten platform (Illumina, San Diego, CA, United States) using 150 bp pair-end sequencing.

Quality control were performed with Trimmomatic (Bolger et al., 2014) according to the following criteria: (a) any reads with adapter; (b) any reads with >50% bases having phred quality <15; (c) any reads with >8% unidentified nucleotides (N). After that, the high-quality reads of each sample were aligned to a reference genome of *S. miltiorrhiza* (Xu et al., 2016) using the SOAP2 (version 2.21) program following the protocols by Li et al. (2009). A maximum of two mismatches (–v 2) was allowed for each read, and those that mapped onto more than one position in the genomic reference sequence were excluded (–r 0). Reads with depths <3 were not counted. The match mode was set to “find the best hits” (–M 4). and the SNPs were obtained based on a maximum likelihood algorithm and filtered by the RADtyping program (Fu et al., 2013). Finally, Quality control was performed using VCF tools (Danecek et al., 2011) to remove SNPs showing a call rate of less than 90%, a minor allele frequency (MAF) of less than 0.05 or significant deviation from Hardy-Weinberg equilibrium (*p* < 0.05).

### Phylogenetic Reconstruction

To infer relationships among samples, we performed phylogenetic analyses with concatenated SNP dataset using maximum likelihood (ML) method. The optimal substitution models for the ML phylogenetic analyses was determined by ModelFinder program (Kalyaanamoorthy et al., 2017) using the Bayesian information criterion (BIC), as implemented in IQ-TREE v2 (Minh et al., 2020). The ML analysis was conducted with IQ-TREE. Five searches were compared to ensure identical topologies. Statistical supports were analyzed using 10,000 replicates of ultrafast bootstrapping (UFBoot: Minh et al., 2013) and 10,000 bootstrap replicates of the Shimodaira/Hasegawa approximate likelihood ratio test (SH-aLRT: Guindon et al., 2010).

### Analyses of Genetic Diversity and Structure

Population genetic statistics, including observed and expected heterozygosity (*H*_*e*_, *H*_o_), nucleotide diversity (π), and Wright’s F statistics of genetic differentiation (*F*_*st*_) and inbreeding coefficient *(F_*IS*_*) were assessed using the “populations” program in Stacks v2.2 (Rochette et al., 2019), and the pairwise statistics between populations were performed using non-parametric tests. Principal component analysis (PCA) and population structure analysis with all SNPs were performed using Plink v1.9 (Purcell et al., 2007) and ADMIXTURE v1.3.0 (Alexander et al., 2009), respectively. Population genetic variation was calculated using an analysis of molecular variance (AMOVA) with packages *adgenet* v2.1 (Jombart and Ahmed, 2011) and *poppr* v2.9 (Kamvar et al., 2015) in R v4.0.3 (R Development Core Team, 2018). Pairwise comparisons of *F*_*st*_ among and within three studied species were estimated. To investigate the correlation between genetic distance and geographic distances, Mantel test was performed using GenAlEx v6.5 (Peakall and Smouse, 2012). Geographic distance between field sampling sites were calculated using *geosphere* v1.5 package, and genetic distances were obtained with *nei.dist* function of *poppr* package.

### Demographic History and Gene Flow

Recent demographic dynamics of *S. miltiorrhiza* was investigated following Liu et al. (2020). A python script easySFS^1^ was used to generate the folded site frequency spectrum (SFS) formatted file with VCF files and sample list file, and demographic history was then inferred using the program Stairway plot v.0.2 (Liu and Fu, 2015). The mutation rate of *S. miltiorrhiza* was set to 2.67 × 10^–8^ per site per generation (unpublished data, based on genomic scale mutation and divergence between *S. miltiorrhiza* and *S. bowleyana*). The generation time was set to 1 year per generation. The program BAYESASS version 3.0.3 (Wilson and Rannala, 2003) was used to examine contemporary gene flow (over the last few generations) and migration rates. Analyses were performed inter-specific and inter-cluster levels separately. We conducted five independent runs using 10^7^ iterations, a burn-in length of 10^6^ and a sampling interval of 10^3^ steps. To reach the recommended acceptance rates, Markov chain Monte Carlo mixing parameter values for migration rates (gene flow), allele frequencies, and inbreeding coefficients were adjusted to 0.50, 0.95, and 0.50, respectively. To confirm convergence, each run was executed with different seed numbers, and trace files were examined for consistent oscillations using Tracer 1.7 (Rambaut et al., 2018).

## Results

### Summary Statistics

A total of 1,097,351,870 raw reads were generated from 151 individuals. After all quality filter, we retained a total of 941,122,629 reads from the initial raw reads, with an average of 6,232,600 reads per sample. The average number of unique tags was 50,031 after removing the unique tags whose sequencing depth was less than 3. Mean sequence depths across all samples was 32×, ranging from 17.77× to 58.41×. After filtering, we obtained 23,928 SNPs in all samples for further analyses.

### Phylogenetic Relationships

**FIGURE 2:**
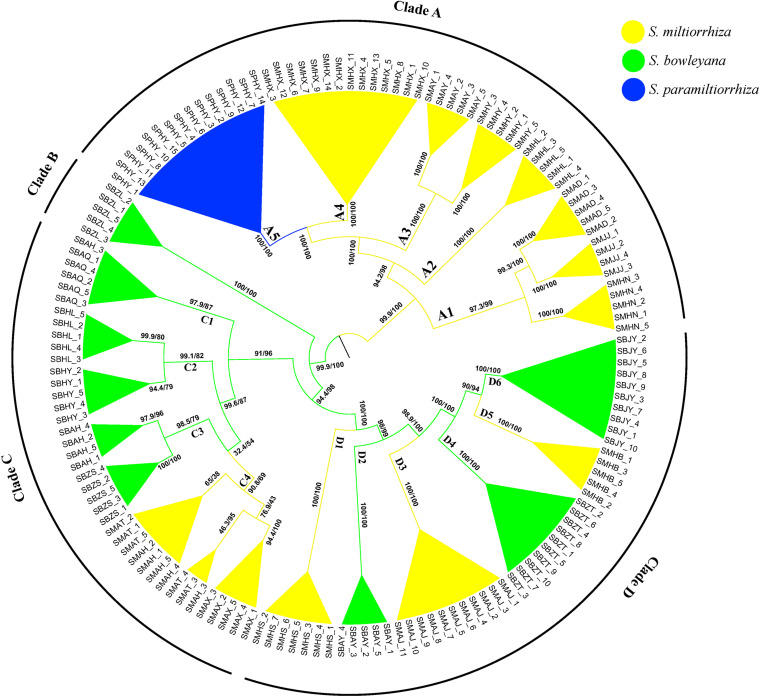
Phylogenetic relationship between *S. miltiorrhiza*, *S. bowleyana*, and *S. paramiltiorrhiza* sampled populations using Maximum Likelihood methods based on SNPs. Bootstrap supports (SH-aLRT/UFBoot values) are indicated at each node. The whole tree can be divided into four major clade (Clade A, B, C, D). Subclade for each species are marked by different color, and the tips label are the individuals name.

The ML phylogeny inferred from TVM + F + R2 model identified four well-supported clades (A–D), with SH-aLRT/UFBoot values displayed above branches (Figure 2). Except several subclades in clade C, all other nodes received robust statistical supports. Clade A includes individuals from 7 populations of *S. miltiorrhiza* and one population of *S. paramiltiorrhiza. Salvia paramiltiorrhiza* population is well-embedded in *S. miltiorrhiza*, forming a monophyly with population SMHX. Clade B includes individuals from a single *S. bowleyana* population (SBZL) of south Zhejiang province. In clade C, SBAQ and one SBAH individual of *S. bowleyana* formed the basal clade C1 (SH-aLRT/UFBoot: 99.6/87). Clade C2 (SBHL and SBHY) is sister to clade C3 + C4. Clade C3 includes SBAH and SBZS of *S. bowleyana* populations is in turn sister to three *S. miltiorrhiza* populations (SMAT, SMAH and SMAX) with weak support (SH-aLRT/UFBoot: 32.4/54). SMHS, SMAJ and SMHB of *S. miltiorrhiza* and SBAY, SBZT and SBJY of *S. bowleyana* showed inter-mixed relationships in clade D.

### Genetic Diversity and Population Structure

In *S. miltiorrhiza* populations, the observed and expected heterozygosity range from 0.0297 to 0.0647 and 0.0251 to 0.0571, respectively. The mean values of π and *F*_*IS*_ are 0.0282 to 0.0641 and −0.0066 to 0.0095, respectively. Significant difference of the genetic parameters were detected among the populations (*H*_*o*_: *p* = 0.000; *H*_*e*_: *p* = 0.000; π: *p* = 0.000; *F*_*IS*_: *p* = 0.000). In *S. bowleyana* populations, *H*_*o*_ and *H*_*e*_ range from 0.0353 to 0.1481 and 0.0322 to 0.0831, respectively. The mean values of π and *F*_*IS*_ are 0.0371 to 0.0890 and −0.1129 to 0.0078. Except *F*_*IS*_, significant difference of the genetic parameters were also detected among the *S. bowleyana* populations (*H*_*o*_: *p* = 0.000; *H*_*e*_: *p* = 0.000; π: *p* = 0.000; *F*_*IS*_: *p* = 0.028). The mean values of *H*_*o*_, *H*_*e*_, π and *F*_*IS*_ in *S. paramiltiorrhiza* are 0.0464, 0.0445, 0.0462, and 0.0018, respectively. High values of private alleles are observed in some populations sampled in the present study (Table 1). Average private alleles in a population are 239 (*S. miltiorrhiza*, highest = 995 in SMHB), 787 (*S. bowleyana*, highest = 4790 in SBZT) and 353 (*S. paramiltiorrhiza*), respectively. The summary statistics of genetic diversity of all three species are given in Table 1.

**TABLE 1:** Population genetic statistics of *S. miltiorrhiza*, *S. bowleyana*, and *S. paramiltiorrhiza* in this study: observed heterozygosity (*H*_*o*_), expected heterozygosity (*H*_*e*_), genetic diversity (π), inbreeding coefficients (*F*_*IS*_).

Taxon	Pop ID	Private alleles	*H*_*o*_	*H*_*e*_	π	*F*_*IS*_
*S. bowleyana*	SBAH	16	0.0591 ± 0.0010	0.0547 ± 0.0009	0.0614 ± 0.0010	0.0058 ± 0.0029
	SBAQ	141	0.0353 ± 0.0009	0.0322 ± 0.0007	0.0371 ± 0.0008	0.0041 ± 0.0048
	SBAY	146	0.0484 ± 0.0010	0.0416 ± 0.0008	0.0471 ± 0.0009	−0.0018 ± 0.0041
	SBHL	13	0.0581 ± 0.0010	0.0545 ± 0.0008	0.0612 ± 0.0010	0.0073 ± 0.0030
	SBHY	11	0.0614 ± 0.0011	0.0552 ± 0.0009	0.0619 ± 0.0010	0.0020 ± 0.0025
	SBJY	1918	0.0508 ± 0.0012	0.0405 ± 0.0009	0.0430 ± 0.0009	−0.0142 ± 0.0105
	SBZL	29	0.0553 ± 0.0010	0.0521 ± 0.0008	0.0587 ± 0.0010	0.0077 ± 0.0035
	SBZS	22	0.0551 ± 0.0010	0.0519 ± 0.0009	0.0587 ± 0.0010	0.0078 ± 0.0035
	SBZT	4790	0.1481 ± 0.0027	0.0831 ± 0.0015	0.0890 ± 0.0016	−0.1129 ± 0.0154
*S. miltiorrhiza*	SMAD	97	0.0408 ± 0.0009	0.0398 ± 0.0008	0.0449 ± 0.0009	0.0095 ± 0.0039
	SMAH	8	0.0489 ± 0.0010	0.0452 ± 0.0008	0.0526 ± 0.0009	0.0083 ± 0.0054
	SMAJ	516	0.0563 ± 0.0010	0.0535 ± 0.0009	0.0566 ± 0.0009	0.0025 ± 0.0085
	SMAT	12	0.0647 ± 0.0011	0.0571 ± 0.0009	0.0641 ± 0.0010	−0.0003 ± 0.0028
	SMAX	39	0.0548 ± 0.0010	0.0493 ± 0.0008	0.0560 ± 0.0010	0.0031 ± 0.0039
	SMAY	55	0.0469 ± 0.0010	0.0408 ± 0.0008	0.0462 ± 0.0009	−0.0009 ± 0.0036
	SMHB	995	0.0456 ± 0.0011	0.0401 ± 0.0008	0.0452 ± 0.0009	−0.0002 ± 0.0053
	SMHL	185	0.0297 ± 0.0009	0.0251 ± 0.0007	0.0282 ± 0.0007	−0.0022 ± 0.0041
	SMHN	77	0.0520 ± 0.0011	0.0450 ± 0.0008	0.0505 ± 0.0009	−0.0027 ± 0.0030
	SMHS	500	0.0430 ± 0.0010	0.0373 ± 0.0008	0.0407 ± 0.0009	−0.0041 ± 0.0054
	SMHX	298	0.0443 ± 0.0009	0.0425 ± 0.0008	0.0443 ± 0.0008	0.0019 ± 0.0090
	SMHY	130	0.0378 ± 0.0010	0.0321 ± 0.0007	0.0362 ± 0.0008	−0.0022 ± 0.0038
	SMJJ	195	0.0370 ± 0.0010	0.0284 ± 0.0007	0.0332 ± 0.0008	−0.0066 ± 0.0030
*S. paramiltiorrhiza*	SPHY	353	0.0464 ± 0.0009	0.0445 ± 0.0008	0.0462 ± 0.0008	0.0018 ± 0.0095

**FIGURE 3:**
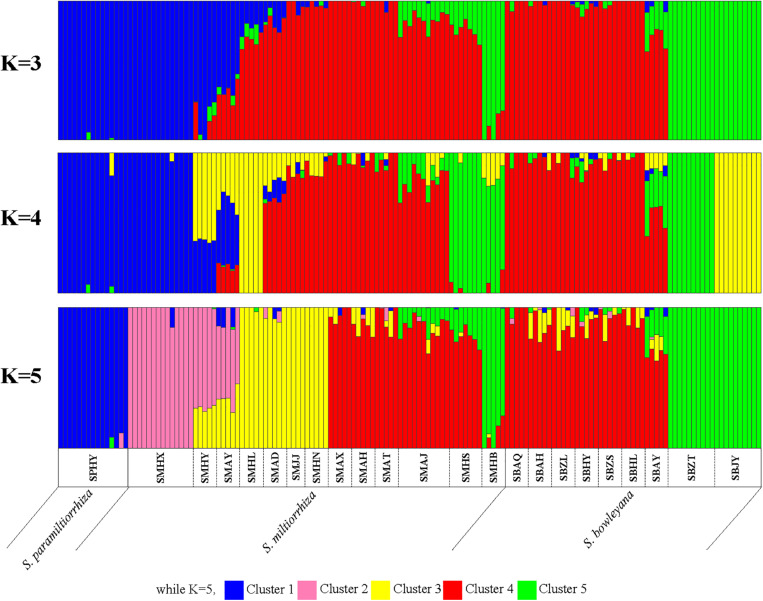
ADMIXTURE bar graphs representing genetic clusters (*K* = 3–5) of samples from the 23 sampling sites divided into three species zones. Each bar represents an individual sample and colors code membership of each individual with assigned cluster. Below the bar, the populations ID and species name were showed.

**FIGURE 4:**
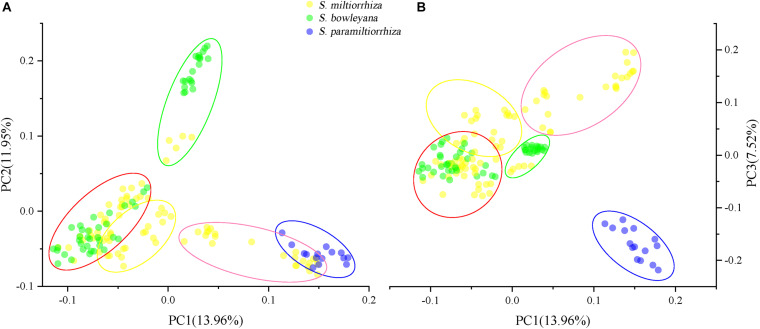
Principal component analysis (PCA) plots showing clustering of samples collected from wild sites. Five ellipse represent the genetic clusters revealed by Admixture analysis (*K* = 5), and the color is consistent with the clusters in Figure 3. **(A)** PCA1-PCA2 plot; **(B)** PCA1-PCA3 plot.

**FIGURE 5:**
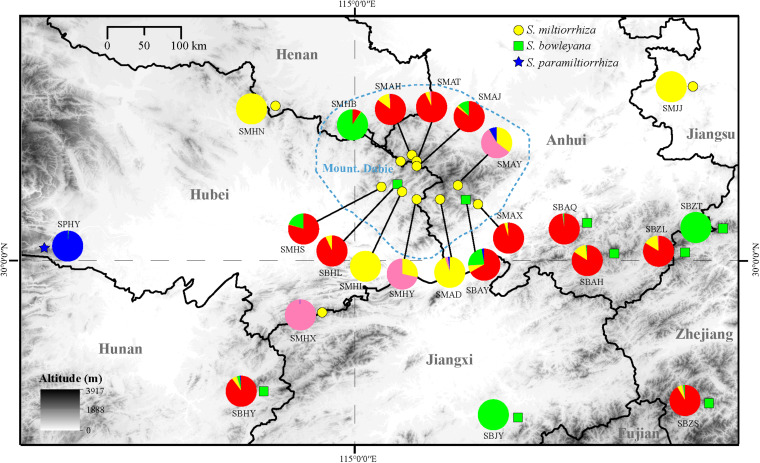
Distribution of the clusters recovered in the Admixture analysis (*K* = 5). The yellow circle, green box and blue five-pointed star represent location of sampled populations of *S. miltiorrhiza*, *S. bowleyana*, and *S. paramiltiorrhiza*, respectively. The blue circled area indicates range of Dabie Mountain. Population codes correspond to those in Supplementary Table 1.

In the Admixture analysis, the CV errors continued to decrease as K increased in value, giving no clear indication of the appropriate K for the study populations. As such, conclusions on population substructuring were based on visual inspection of the admixture plots (Figure 3) and PCA plots (Figure 4). A graphic representation of cluster structure analysis is depicted in Figure 3 with K from 3 to 5. The species and the genotype boundary between groups is clearer at *K* = 5. *Salvia paramiltiorrhiza* is dominated by cluster 1. Four major clusters were observed in *S. miltiorrhiza* (cluster 2–5). It also indicated that cluster 4 and 5 were shared by six populations of *S. miltiorrhiza* (SMAX, SMAH, SMAT, SMAJ, SMHS, and SMHB) and *S. bowleyana* (Figure 3, *K* = 5). Different degrees of inter/intra-specific admixtures were observed in the studied populations. Further insights into population structure were obtained by plotting the five genetic clusters on the map (Figure 5). Geographical distribution of the genetic clusters at population level did not show conspicuous genetic structure.

For PCA plots, PC1, PC2, and PC3 explained approximately 33.43% of the total variation. When we grouped the samples according to the five clusters in Admixture analysis, cluster 1 and 2 overlapped to each other in PC1-PC2 plot (Figure 4A), while showed a distant relationship in PC1-PC3 plot (Figure 4B). Cluster 3, a genetic group from *S*. *miltiorrhiza* showed close relationship with cluster 4 in both plots. In cluster 4, individuals of *S. miltiorrhiza* and *S. bowleyana* mixed together (Figures 4A,B). Cluster 5 showed isolated relationships with other clusters. It is also noted that while share the same cluster 5, individuals of *S. miltiorrhiza* (SMHB) show a clear distance with *S. bowleyana* populations (SBZT and SBJY).

AMOVA analyses was performed among species and within each species separately. For within-species analysis, we grouped the populations according to the five ancestry clusters recovered by Admixture analysis. The results revealed that inter-specific differences account for 13.12% of the genetic variation. Most of genetic variation is due to the differences within populations (49.78%) (Table 2). The same pattern was observed in *S. miltiorrhiza* analyses, that high percentage of the genetic variance (65.52%) came from the differences within populations. In *S. bowleyana*, most of the genetic variation (56.36%) is due to differences between populations within clusters. Pairwise genetic differentiation (*F*_*st*_) between samples from sampling sites were different significantly, as shown in Supplementary Table 2, and the results are consistent with the genetic variation between populations by AMOVA. Mantel test failed to detect a significant isolation by distance (IBD) based on the geographical distance and genetic divergence matrices in both species (*S. miltiorrhiza*: *R*^2^ = 0.0013, *p* = 0.460; *S. bowleyana*: *R*^2^ = 0.0174, *p* = 0.450).

**TABLE 2:** Analysis of molecular variance (AMOVA) showing genetic variation among and within three studied species from collection sites.

Source of variation	Sum Sq	Variance components	% of variation	*P*-value	F-Statistics
**Interspecific comparison**					
Among three species	108340.00	704.14	13.12	0.076	0.1312
Among populations within species	295574.20	1989.97	37.09	0.001	0.4270
Within populations	341878.20	2670.92	49.78	0.001	0.5022
***Salvia miltiorrhiza***					
Among Clusters	52783.73	566.43	15.35	0.001	0.1535
Among populations within Clusters	62582.16	706.21	19.13	0.001	0.2260
Within populations	162008.66	2418.04	65.52	0.001	0.3448
***Salvia bowleyana***					
Among Clusters	74212.78	1346.46	16.46	0.197	0.1646
Among populations within Clusters	199950.89	4609.83	56.36	0.001	0.6747
Within populations	102235.60	2222.51	27.17	0.001	0.7283

### Migration Rates and Gene Flow Analysis

For inter-specific migrations, we detected significant gene flow from *S. bowleyana* to *S. miltiorrhiza* (21.45%) estimated in BAYESASS (Table 3). It is consistent with the Admixture results that several *S. miltiorrhiza* populations shared clusters with *S. bowleyana*. The second highest but not significant migration was inferred from *S. paramiltiorrhiza* to *S. miltiorrhiza*. For inter-cluster runs, overall low levels of contemporary gene flow were revealed. The highest migration rates were inferred from the cluster 5 to 4 (6.44%), cluster 1 to 2 (3.23%), and cluster 3 to 4 (2.56%). Cluster 4 received comparatively higher gene flows from other clusters. This is also observed in Admixture analyses that most individuals from cluster 4 showed differential admixture levels with other clusters.

**TABLE 3:** Posterior means of contemporary migration rates among species and genetic clusters estimated by BAYESASS (values in brackets are standard deviations, and mean values more than 0.1 are considered significant).

Populations	Migration rates
SB→SM	**0.2145** (0.0219)
SB→SP	0.0060 (0.0058)
SM→SB	0.0156 (0.0076)
SM→SP	0.0040 (0.0040)
SP→SB	0.0175 (0.0165)
SP→SM	0.0250 (0.0217)
cluster1→cluster4	0.0158 (0.0152)
cluster1→cluster2	0.0323 (0.0211)
cluster1→cluster3	0.0165 (0.0157)
cluster1→cluster5	0.0158 (0.0152)
cluster2→cluster4	0.0227 (0.0152)
cluster2→cluster3	0.0115 (0.0110)
cluster2→cluster5	0.0115 (0.0113)
cluster2→cluster1	0.0118 (0.0115)
cluster3→cluster4	0.0256 (0.0175)
cluster3→cluster2	0.0134 (0.0131)
cluster3→cluster5	0.0134 (0.0127)
cluster3→cluster1	0.0134 (0.0128)
cluster4→cluster2	0.0046 (0.0045)
cluster4→cluster3	0.0191 (0.0092)
cluster4→cluster5	0.0238 (0.0101)
cluster4→cluster1	0.0048 (0.0047)
cluster5→cluster4	0.0644 (0.0235)
cluster5→cluster2	0.0109 (0.0105)
cluster5→cluster3	0.0106 (0.0105)
cluster5→cluster1	0.0107 (0.0102)

*SM: *S. miltiorrhiza*; SB: *S. bowleyana*; SP: *S. paramiltiorrhiza*. The bold value indicates strong signal of contemporary migration.*

### Effective Population Size and Demographic History

**FIGURE 6:**
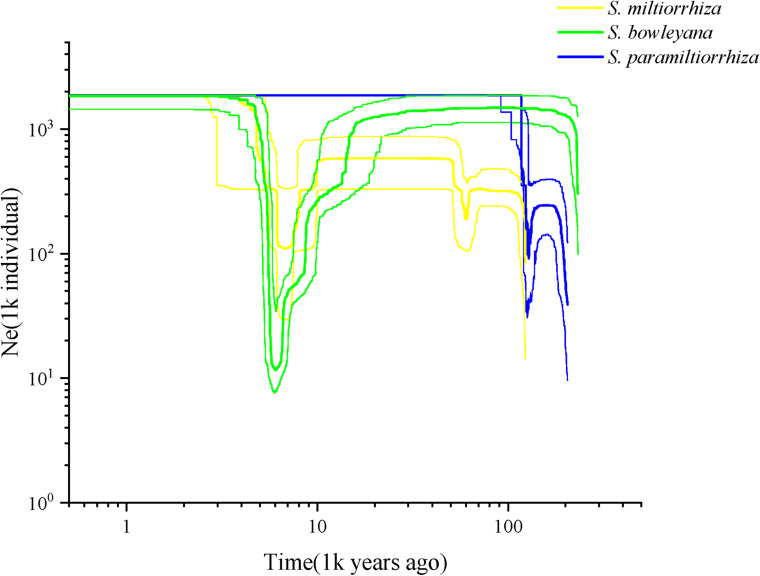
Reconstructed demographic history of *S. miltiorrhiza* (yellow), *S. bowleyana* (green), and *S. paramiltiorrhiza* (blue). Stairway plot showing historical changes in effective population size for three species population with generation time of 1 year.

Effective population size and demographic history of three studied species were inferred based on SNP frequency spectra and displayed on stairway plots (Figure 6). For *S. miltiorrhiza*, an initial shallow population contraction occurred at ca. 60 kya (thousand years ago), after which followed a population expansion and then a flat period until 8 kya. Then, a second more severe population bottleneck occurred during ca. 8–6 kya, which followed another population expansion and then a flat growth period maintained to present. For *S. bowleyana* populations, a deep population bottleneck was also detected around 8–6 kya, which followed a similar population dynamics as *S. miltiorrhiza*. *Salvia paramiltiorrhiza* experienced an early population contraction at ca. 104 kya, and then rapidly expanded and maintained its population size to present.

## Discussion

Preserving the biological and genetic diversity from wild plant species were considered as an essential and effective way of conservation and sustainable use of medicinal plants (Hamilton, 2008; Chen et al., 2016; Vining et al., 2020). Therefore, it is important to characterize genetic diversity and population structure of targeted species and its relatives, especially threatened ones (Lee et al., 2018, 2020). The genetic diversity in three studied species are relatively high compare to other medicinal herbs, for example, *Coptis* species (Wang et al., 2020), *Valeriana officinalis* (Boczkowska et al., 2020), *Aconitum austrokoreense* (Lee et al., 2018). Especially for *S. paramiltiorrhiza*, characterized by yellow flower and longer tube without hair annulus, high genetic diversity was observed (0.0462) despite its small population size and restricted distribution. This could be due to its outcrossing behavior and possible historical introgression with *S. miltiorrhiza*, which is supported by PCA plot (Figure 4A). Although phylogenetic analysis indicated that *S. paramiltiorrhiza* is nested within *S. miltiorrhiza* populations (Figure 2), Admixture analysis (Figure 3, *K* = 5) and PCA plot (Figure 4B) revealed that it’s a distinct cluster to *S. miltiorrhiza* and *S. bowleyana* populations. Pairwise *F*_*st*_ also indicates that SPHY, the *S. paramiltiorrhiza* population, is significantly differentiated from all other studied populations of *S. miltiorrhiza* and *S. bowleyana* (Supplementary Table 2). Geographically, *S. paramiltiorrhiza* is isolated from other sampled populations (Figure 5). However, admixture from *S. paramiltiorrhiza* to *S. miltiorrhiza* were detected in population SMHX, SMAY, and SMAD. A more extensive field examination is needed to confirm whether coexistence of *S. miltiorrhiza* is occurred in its distribution range.

Relationships between *S. miltiorrhiza* and *S. bowleyana* indicated a possible complex history. Morphologically, *S. bowleyana* can be distinguished from *S. miltiorrhiza* by its brownish root and yellow to pink upper corolla lips (Figure 1). Genetic analyses indicated that six populations (SMAX, SMAH, SMAT, SMAJ, SMHS, SMHB) of *S. miltiorrhiza* shared cluster 4 and 5 with *S. bowleyana* populations in Admixture analysis, which is supported by phylogenetic analysis (clade C and D, Figure 2). All of these six populations distributed in the Dabie Mountain area, which overlaps the northern distribution range of *S. bowleyana* (Figure 5). On one hand, we assumed that the frequent gene flows and possible hybridization might contributed to the mixture between these two species. The species used in this study were carefully identified, nonetheless, one could actually be hybrids with morphological similarity to one of the parent species. Although unidirectional migration of gene flow is detected from *S. bowleyana* to *S. miltiorrhiza* in BAYESASS analyses, we observed reciprocal admixtures between populations of these two species (Figure 3), which is also indicated by the low inter-specific differentiation (0.1312) by AMOVA (Table 2). The weakly supported subclades in clade C might indicates the influence of partially contradict informative SNPs introduced by the gene flow between populations of these two species. Moreover, *S. miltiorrhiza* and *S. bowleyana* blossom at the same time and share pollinators (field observation), which indicate that the reproductive barriers between them might be weak to some extent, leading to inter-specific gene flow. On the other hand, since these two species occur in partial allopatry, the undifferentiated genetic divergence could also be a result of retention of ancestral polymorphism, as incomplete lineage sorting could lead to a similar phenomenon which was observed in many other closely related taxa (Alexander et al., 2017; Goetze et al., 2017; Cerca et al., 2021). Pairwise *F*_*st*_ estimates between SMAX, SMAH, SMAT, SMHB from *S. miltiorrhiza* and population SBHL, SBHY, SBZS, SBZL, SBAH, SBAQ of *S. bowleyana* were not statistically significant (Supplementary Table 2). In phylogenetic analysis, clade B & C, dominated by populations from cluster 4, is sister to clade D, which is dominated by populations from cluster 5. Nonetheless, PCA analysis suggested a closer distance of the mixed cluster 4 and cluster 3 (*S. miltiorrhiza* populations in clade A). This indicated that both species could potentially contributed to the ancestry of cluster 4. It is also noteworthy that populations of *S. miltiorrhiza* (SMHB, SMHS, SMAJ) and *S. bowleyana* (SBZT, SBJY, SBAY) in cluster 5 did not mixed to each other as showed in PCA plot (Figure 4), despite sharing the same genetic ancestry revealed by Admixture analysis. Geographically, population SMHB is distantly located to SBZT/SBJY, indicating low recent gene flow may occur between them. It suggests a retention of ancestral polymorphism of cluster 5 in both species. The AMOVA revealed different genetic differentiation scenarios of *S. miltiorrhiza* and *S. bowleyana*. Differentiation among populations within clusters of *S. bowleyana* is high, and composed most of the genetic variation (56.36%). However, in *S. miltiorrhiza*, most of variation were detected within populations, and low genetic differentiation were observed in all group settings. This might imply that in spite of frequent gene follow, these two species might experienced differential evolutionary history. Further studies on *S. miltiorrhiza* and *S. bowleyana* at a larger population scale will be necessary to clarify the boundary between these two species.

Our analyses provided insights into the distribution of genetic diversity in *S. miltiorrhiza*, *S. bowleyana*, and *S. paramiltiorrhiza*, revealing that populations located in the Dabie Mountain range harbor the greatest amount of diversity (Figure 5). This area could potentially serve as a connection between *S. miltiorrhiza* from the north and *S. bowleyana* from the south. They might have experienced continuous gene flow over time, increasing toward the present day. According to the demographic history of *S. miltiorrhiza* and *S. bowleyana* illustrated by stairway plot, they experienced a contemporary bottleneck occurred at 8–6 kya (Figure 6), which was consistent with the 8.2 k event of global cooling and population reduction in the Holocene epoch (Alley and Agustsdottir, 2005; Wicks and Mithen, 2014), which imply that *S. miltiorrhiza* and *S. bowleyana* may have shared refugia during climatic oscillations, where hybridization occurred. *Salvia miltiorrhiza* experienced an additional population contraction occurred at ca. 60 kya, which was consistent with the abrupt climate change and ecological turnover in late Pleistocene glaciation period (Finlayson and Carrión, 2007; Astakhov, 2008). *Salvia bowleyana* and *S. paramiltiorrhiza* might avoid the contraction during the glaciation due to their further south distribution range compare to *S. miltiorrhiza*. Therefore, genetic exchange could also occur among them when *S. miltiorrhiza* retreated further south during that period. Population expansion of both *S. miltiorrhiza* and *S. bowleyana* took place during 6–4 kya, after which the population size of both species reached a stable status to present. No recent reduction in population size was observed based on genetic data. However, due to recent excessive excavation and habitat destruction, the wild populations of *S. miltiorrhiza* and its relatives reduced drastically. This could be explained by that the populations we sampled in the study were mostly collected in the nature reserves, where human activities are rare and restricted. Moreover, the population destruction started just decades ago, as such the role of genetic drift will be limited over such a short period of time (Johnson et al., 2018). Otherwise, the frequent gene flow among the species could also contributed to their maintenance of genetic diversity.

Our study suggests a complex scenario of inter/intra-specific evolutionary history of among *S. miltiorrhiza*, *S. bowleyana*, and *S. paramiltiorrhiza*. Incomplete lineage sorting, repeated gene flow, as well as localized events of hybridization together might have shaped the current genetic structure and intermixed relationship among these three closely related species. The molecular data indicates that *S. paramiltiorrhiza* exhibits a unique lineage after comparing with *S. miltiorrhiza* and *S. bowleyana* populations. Additionally, several novel compounds has been discovered from the roots of *S. paramiltiorrhiza* (Sun et al., 1991, 1992; Lin and Chang, 2000). For *S. bowleyana*, a complex inter-specific interaction history between *S. miltiorrhiza* was revealed. It leads to a better understanding of the evolutionary history and dynamics of this species complex group. As relatives with *S. miltiorrhiza*, *S. paramiltiorrhiza* and *S. bowleyana* could serve as valuable wild germplasm resources to improve the quality of *S. miltiorrhiza* or develop novel breedings. Protection of the wild relatives of the targeted plant species will greatly enhance the scale of the genetic diversity and increase resilience to the agricultural system through breeding practice (Ford-Lloyd et al., 2011; Tyack et al., 2020). Therefore, we suggest that wild populations of *S. paramiltiorrhiza* and *S. bowleyana* should be considered together as whole with *S. miltiorrhiza* in wild germplasm conservation practice. For *S. paramiltiorrhiza*, on the basis of its restricted geographical distribution and threatened condition, we suggested that *in situ* conservation efforts should be made such as establishing protected areas and population recovering in natural habitat. Our study also revealed Dabie Mountain as a genetic diversity center and corridor of these three species. This region should be prioritized as a potential area of conservation, which would ensure the survival and maintain the evolutionary potential of the group. In the meantime, seed bank construction and *ex situ* conservation through botanical gardens are also recommended, as it also effectively preserve the biological and genetic diversity of *S. miltiorrhiza*, *S. bowleyana* and *S. paramiltiorrhiza* for sustainable usage. We hope this study would provide a guidance to the conservation of other medicinal plants when species history are complex and wild relatives are involved.

## Data Availability Statement

The raw sequencing data has been successfully uploaded to SRA in NCBI, https://www.ncbi.nlm.nih.gov/Traces/study/?acc=PRJNA716793.

## Author Contributions

Y-KW and Z-CQ conceived and designed the experiments and revised the manuscript. Y-BH and H-WX carried out investigation and collected materials. XZ, Z-CZ, J-JW, Z-CQ, and Y-KW analyzed the data and wrote the manuscript. Y-KW and Z-CQ acquired the funding. All authors contributed to the article and approved the submitted version.

## Conflict of Interest

The authors declare that the research was conducted in the absence of any commercial or financial relationships that could be construed as a potential conflict of interest.

## Footnotes

1 https://github.com/isaacovercast/easySFS
