# Supplementary material for: Conservation Genomics of Wild Red Sage (Salvia miltiorrhiza) and Its Endangered Relatives in China: Population Structure and Interspecific Relationships Revealed From 2b-RAD Data
Source: Front Genet. 2021 May 11;12:688323. doi: 10.3389/fgene.2021.688323 (PMC8144715; doi:10.3389/fgene.2021.688323)
Supplement: Supplementary file 1 [file Data_Sheet_1.doc]

Table S1. Collection information of *S.miltiorrhiza*, *S. bowleyana* and *S. paramiltiorrhiza* in this study. Voucher specimens were deposited in Herbarium of Chenshan Botanical Garden (CHS), Shanghai, China.

| Taxon | Population code | Sample size | Locality | Longitude | Latitude | Altitude | Voucher |
| --- | --- | --- | --- | --- | --- | --- | --- |
| *S. bowleyana* | SBAH | 5 | Huangshan, Anhui | 118°08'09"E | 30°05'29"N | 796m | CHS_S0344 |
|  | SBAQ | 5 | Qingyang, Anhui | 117°47'54"E | 30°29'24"N | 453m | CHS_S0346 |
|  | SBAY | 5 | Yuexi, Anhui | 116°19'26"E | 30°45'14"N | 487m | CHS_S0162 |
|  | SBHL | 5 | Luotian, Hubei | 115°28'58"E | 30°56'58"N | 250m | CHS_S0270 |
|  | SBHY | 5 | Liuyang, Hunan | 113°51'35"E | 28°24'09"N | 158m | CHS_S0298 |
|  | SBJY | 10 | Yingtan, Jiangxi | 116°57'29"E | 28°05'35"N | 94m | CHS_S0568 |
|  | SBZL | 5 | Lin'an, Zhejiang | 118°59'43"E | 30°06'17"N | 229m | CHS_S0334 |
|  | SBZS | 5 | Songyang, Zhejiang | 119°17'31"E | 28°16'09"N | 723m | CHS_S0299 |
|  | SBZT | 10 | Lin'an, Zhejiang | 119°28'35"E | 30°23'45"N | 945m | CHS_S0603 |
| *S. miltiorrhiza* | SMAD | 5 | Yuexi, Anhui | 115°59'49"E | 30°46'07"N | 265m | CHS_S0433 |
|  | SMAH | 5 | Jinzhai, Anhui | 115°39'39"E | 31°18'15"N | 636m | CHS_S0263 |
|  | SMAJ | 11 | Jinzhai, Anhui | 115°44'35"E | 31°11'16"N | 640m | CHS_S0496 |
|  | SMAT | 5 | Jinzhai, Anhui | 115°44'24"E | 31°13'26"N | 767m | CHS_S0264 |
|  | SMAX | 5 | Yuexi, Anhui | 116°28'19"E | 30°42'17"N | 306m | CHS_S0172 |
|  | SMAY | 5 | Yuexi, Anhui | 116°13'35"E | 30°55'39"N | 471m | CHS_S0347 |
|  | SMHB | 5 | Luotian, Hubei | 115°31'13"E | 31°13'30"N | 262m | CHS_S0498 |
|  | SMHL | 5 | Luotian, Hubei | 115°32'26"E | 30°51'03"N | 349m | CHS_S0353 |
|  | SMHN | 5 | Xinyang, Henan | 113°59'12"E | 31°53'49"N | 130m | CHS_S0359 |
|  | SMHS | 7 | Luotian, Hubei | 115°17'22"E | 30°54'44"N | 140m | CHS_S0497 |
|  | SMHX | 14 | Xianning, Hubei | 114°33'51"E | 29°22'39"N | 405m | CHS_S0494 |
|  | SMHY | 5 | Yingshan, Hubei | 115°43'15"E | 30°45'48"N | 189m | CHS_S0349 |
|  | SMJJ | 4 | Jurong, Jiangsu | 119°05'18"E | 32°08'12"N | 123m | CHS_S0427 |
| *S. paramiltiorrhiza* | SPHY | 15 | Yichang, Hubei | 111°11'03"E | 30°10'48"N | 211m | CHS_S0491 |

Table S2. Genetic distances (*F_st_* values, above diagonal) and geographic distances (km, below diagonal) between populations from different sample sites.

|  | SBAY | SMAX | SMAH | SMAT | SBHL | SBHY | SBZS | SBZL | SBAH | SBAQ | SMAY | SMHY | SMHL | SMHN | SMJJ | SMAD | SPHY | SMHX | SMAJ | SMHS | SMHB | SBJY | SBZT |
| --- | --- | --- | --- | --- | --- | --- | --- | --- | --- | --- | --- | --- | --- | --- | --- | --- | --- | --- | --- | --- | --- | --- | --- |
| SBAY | 0 | 0.150 | 0.161 | 0.136 | 0.141 | 0.142 | 0.161 | 0.157 | 0.143 | 0.267 | 0.222 | 0.273 | 0.313 | 0.198 | 0.293 | 0.225 | **0.188** | **0.202** | **0.163** | **0.254** | 0.379 | **0.462** | **0.589** |
| SMAX | 18.77 | 0 | 0.101 | 0.085 | 0.091 | 0.091 | 0.114 | 0.118 | 0.099 | 0.201 | 0.176 | 0.223 | 0.258 | 0.152 | 0.231 | 0.176 | **0.156** | **0.167** | **0.141** | 0.219 | 0.344 | **0.428** | **0.569** |
| SMAH | 90.51 | 103.82 | 0 | 0.076 | 0.083 | 0.085 | 0.106 | 0.113 | 0.090 | 0.207 | 0.175 | 0.225 | 0.262 | 0.148 | 0.236 | 0.176 | **0.148** | **0.161** | **0.138** | 0.222 | 0.350 | **0.438** | **0.575** |
| SMAT | 73.80 | 86.05 | 18.59 | 0 | 0.071 | 0.072 | 0.091 | 0.099 | 0.077 | 0.173 | 0.150 | 0.187 | 0.219 | 0.127 | 0.194 | 0.150 | **0.140** | **0.149** | **0.130** | **0.193** | 0.309 | **0.393** | **0.544** |
| SBHL | 83.47 | 102.00 | 58.06 | 60.15 | 0 | 0.071 | 0.091 | 0.100 | 0.078 | 0.181 | 0.155 | 0.196 | 0.227 | 0.130 | 0.200 | 0.156 | **0.143** | **0.153** | **0.134** | **0.198** | 0.316 | **0.402** | **0.549** |
| SBHY | 380.56 | 394.65 | 392.96 | 396.25 | 336.26 | 0 | 0.092 | 0.101 | 0.080 | 0.180 | 0.152 | 0.194 | 0.224 | 0.130 | 0.199 | 0.155 | **0.143** | **0.154** | **0.133** | 0.196 | 0.316 | **0.401** | **0.550** |
| SBZS | 400.45 | 387.01 | 490.62 | 473.05 | 472.53 | 572.99 | 0 | 0.114 | 0.091 | 0.203 | 0.173 | 0.218 | 0.254 | 0.147 | 0.226 | 0.173 | **0.157** | **0.167** | **0.146** | 0.218 | 0.338 | **0.428** | **0.565** |
| SBZL | 262.73 | 245.12 | 344.46 | 325.87 | 345.36 | 555.33 | 192.13 | 0 | 0.097 | 0.197 | 0.160 | 0.204 | 0.230 | 0.134 | 0.212 | 0.161 | **0.145** | **0.156** | **0.124** | 0.209 | 0.329 | **0.416** | **0.560** |
| SBAH | 195.58 | 178.94 | 281.30 | 262.89 | 276.73 | 487.22 | 220.99 | 72.37 | 0 | 0.177 | 0.152 | 0.193 | 0.226 | 0.128 | 0.201 | 0.152 | **0.142** | **0.151** | **0.132** | **0.197** | 0.315 | **0.401** | **0.549** |
| SBAQ | 152.18 | 133.63 | 228.57 | 209.99 | 235.61 | 491.57 | 289.50 | 117.32 | 69.45 | 0 | 0.276 | 0.339 | 0.384 | 0.244 | 0.361 | 0.276 | **0.229** | **0.239** | **0.200** | 0.329 | 0.447 | **0.528** | **0.628** |
| SMAY | 23.14 | 31.00 | 73.15 | 55.14 | 80.63 | 395.05 | 417.98 | 273.08 | 208.49 | 159.01 | 0 | 0.199 | 0.288 | 0.186 | 0.282 | 0.205 | **0.153** | **0.146** | **0.181** | 0.278 | 0.401 | **0.485** | **0.601** |
| SMHY | 56.79 | 75.40 | 59.72 | 53.32 | 26.73 | 347.60 | 448.34 | 318.70 | 250.3 | 208.97 | 56.09 | 0 | 0.371 | 0.243 | 0.359 | 0.267 | **0.202** | **0.205** | **0.215** | **0.339** | 0.460 | **0.535** | **0.631** |
| SMHL | 69.86 | 86.44 | 31.78 | 29.14 | 31.28 | 367.12 | 469.03 | 331.40 | 265.23 | 218.13 | 59.19 | 28.07 | 0 | 0.271 | 0.405 | 0.296 | **0.241** | **0.250** | 0.234 | **0.379** | 0.493 | **0.565** | **0.650** |
| SMHN | 260.42 | 274.42 | 170.60 | 188.66 | 195.77 | 443.67 | 660.88 | 512.02 | 451.17 | 394.90 | 243.72 | 216.02 | 192.55 | 0 | 0.241 | 0.171 | **0.165** | **0.175** | **0.168** | **0.251** | 0.376 | 0.463 | **0.590** |
| SMJJ | 308.07 | 290.72 | 348.73 | 333.32 | 381.50 | 677.68 | 388.67 | 205.21 | 229.21 | 191.85 | 302.17 | 358.22 | 354.42 | 480.42 | 0 | 0.260 | **0.224** | **0.236** | **0.220** | 0.345 | 0.474 | 0.547 | 0.634 |
| SMAD | 23.81 | 42.51 | 74.69 | 60.58 | 59.73 | 365.30 | 419.74 | 285.98 | 218.12 | 175.98 | 29.77 | 33.02 | 49.31 | 241.76 | 329.25 | 0 | **0.178** | **0.186** | **0.184** | **0.281** | 0.406 | **0.490** | **0.603** |
| SPHY | 476.74 | 495.41 | 414.93 | 431.43 | 393.53 | 361.69 | 831.52 | 734.83 | 663.69 | 628.84 | 472.90 | 420.01 | 414.68 | 311.44 | 762.90 | 452.93 | 0 | **0.136** | **0.182** | **0.245** | **0.356** | **0.453** | **0.596** |
| SMHX | 210.76 | 228.27 | 200.25 | 206.51 | 146.95 | 196.99 | 524.66 | 443.22 | 370.85 | 350.75 | 218.69 | 164.46 | 178.15 | 257.03 | 518.83 | 190.16 | 307.10 | 0 | **0.191** | **0.261** | **0.372** | **0.466** | **0.606** |
| SMAJ | 69.74 | 82.40 | 21.56 | 4.51 | 57.46 | 393.23 | 469.33 | 323.14 | 259.75 | 207.53 | 51.62 | 49.28 | 26.21 | 192.10 | 333.24 | 56.11 | 432.19 | 204.18 | 0 | **0.223** | **0.312** | **0.404** | **0.562** |
| SMHS | 95.57 | 113.94 | 57.68 | 63.63 | 12.83 | 335.30 | 485.28 | 357.84 | 289.39 | 247.55 | 91.21 | 39.15 | 36.77 | 184.28 | 390.51 | 71.96 | 382.02 | 143.19 | 61.72 | 0 | **0.439** | **0.522** | **0.629** |
| SMHB | 88.68 | 104.42 | 19.88 | 29.85 | 39.11 | 373.11 | 488.57 | 348.60 | 283.34 | 234.21 | 75.53 | 46.44 | 19.93 | 172.74 | 363.03 | 69.02 | 402.51 | 180.47 | 29.84 | 37.81 | 0 | **0.491** | **0.613** |
| SBJY | 283.94 | 280.99 | 365.00 | 352.43 | 323.45 | 335.91 | 237.56 | 272.30 | 227.38 | 274.70 | 306.95 | 307.65 | 334.97 | 517.85 | 456.39 | 291.92 | 621.91 | 319.42 | 347.94 | 334.14 | 354.08 | 0 | **0.643** |
| SBZT | 306.50 | 288.17 | 382.86 | 364.35 | 389.93 | 611.93 | 210.49 | 56.93 | 126.31 | 155.02 | 314.03 | 363.21 | 373.15 | 546.38 | 178.81 | 330.20 | 782.19 | 495.31 | 362.07 | 402.06 | 389.15 | 326.23 | 0 |

Bold indicates P < 0.001. Significance levels were obtained after 1,000 permutations.
